# Immediate Effects of Multiple Ischemic Compression Applications on Pain Sensitivity and Biomechanical Properties of Myofascial Trigger Points

**DOI:** 10.3390/clinpract15070125

**Published:** 2025-07-01

**Authors:** Sebastian Szajkowski, Jarosław Pasek, Grzegorz Cieślar

**Affiliations:** 1 Faculty of Medical Sciences, Warsaw Medical Academy of Applied Sciences, 8 Rydygiera St., 01-793 Warszawa, Poland; sebastianszajkowski@wp.pl; 2 Collegium Medicum im dr Władysława Biegańskiego, Jan Długosz University in Częstochowa, 13/15 Armii Krajowej St., 42-200 Częstochowa, Poland; 3 Department of Internal Medicine, Angiology and Physical Medicine, Faculty of Medical Sciences in Zabrze, Medical University of Silesia in Katowice, 15 Stefana Batorego St., 41-902 Bytom, Poland; cieslar1@o2.pl

**Keywords:** myofascial trigger points, pain threshold, pain perception, ischemic compression, myotonometry

## Abstract

**Background:** Myofascial trigger points (MTrPs) are hyperirritable spots within taut bands of skeletal muscle fibers, often developing in overloaded muscles. Ischemic compression (IC) is a frequently used therapeutic technique for MTrP treatment. **Material and Methods:** Seventy-nine participants with MTrPs in the upper trapezius muscle were included. Three IC protocols were used. In group 1, the compression force was increased once; in group 2, twice; and in group 3, three times—each time up to the pain threshold, then held constant until the pain subsided. Evaluations included pressure pain threshold (PPT), pressure pain perception (PPP), and myotonometric measurements. **Results:** PPT values increased significantly in group 2 (*p* = 0.009) and group 3 (*p* = 0.009), while PPP values decreased significantly in both groups (group 2: *p* = 0.016; group 3: *p* = 0.041) post-intervention. Group 1 showed a significant reduction in muscle tone (*p* < 0.001), and group 2 in muscle stiffness (*p* = 0.036). Muscle elasticity significantly improved in all groups: group 1 (*p* = 0.022), group 2 (*p* = 0.001), and group 3 (*p* = 0.042). **Conclusions:** IC applied with a constant force at the individual’s pain perception threshold effectively elevates the pain threshold and enhances the biomechanical parameters of muscle fibers in the trigger point area.

## 1. Introduction

Myofascial trigger points (MTrPs) develop in overloaded, tense, and shortened muscles. They are hyperirritable points or spots in the fiber band of a skeletal muscle. The Literature data indicate that chronic static loads of an isometric nature and eliciting eccentric muscle contraction cause long-term pressure on capillaries, impairing capillary circulation [1,2,3]. As a result of hypoxia and acidification, the so-called “energy crisis” develops, with deficiency of adenosine triphosphate (ATP) [4]. It is believed that mechanisms of peripheral and central nervous system sensitization have a role to play in the development of pain and of local contracture along a section of muscle fibers, typically detected on palpation. An inflamed and neuro-chemically altered environment of MTrPs is as follows: elevated level of bradykinin, substance P, and calcitonin gene-related peptide (CGRP) [5], as well as reduced pH level, which sensitizes neurons and afferent fibers, intensifying the nociceptive signal [6].

The therapeutic procedure of applying ischemic compression (IC) consists of a slow increase of the initially painless pressure exerted on the MTrPs, until the pain perception threshold is reached. The compression is then maintained until the touch and pressure receptors are habituated [7]. At the same time, ischemia develops as a result of compression and vasoconstriction. As a result of temporary disruption of homeostasis, secondary hyperemia occurs in the tissue that is subject to therapy, as a result of reflexive dilatation of blood vessels.

Thus, the therapeutic effect is connected with mechanical and reflexive action: modulation of the sensation of pain, which functions in accordance with the control gate theory developed by Wall and Melzack [8], and vasodilatation [9,10]. The gating mechanism in the spinal dorsal horn is affected by relative activity in the large-diameter (A-Beta nerve fibers) and small-diameter (A-Delta and C nerve fibers) range, with the former inhibiting transmission (closing the gate) and the latter facilitating transmission (opening the gate). Latent MTrPs are used in the study. On the one hand, such an attempt does not represent a population typical for everyday clinical practice; on the other hand, however, the authors wanted to limit the influence of differently intensified sensitization of active threshold points upon pain assessment, which in itself is subjective and which determines the efficiency of IC.

Although the subjective examination method by means of palpation remains the most popular method for diagnosing MTrPs as well as assessing pain sensation and muscle tension, there are also objective methods available for quantitative assessment in clinical practice [11,12]; in the opinion of the authors of this report, they should be applied more extensively, both in diagnostics and in monitoring the progress of treatment, due to the high repeatability of the results of measurements taken. Myotonometry is one of the simplest, most inexpensive methods applicable, being also mobile; all in all, this enables measurements of biomechanical properties of muscles in vivo. It can also find application in the case of MTrPs [13,14]. Pressure pain sensitivity, which accompanies MTrPs, in turn, can be measured by either the Pressure Pain Threshold (PPT) method or by Pressure Pain Perception (PPP) [15].

There is evidence supporting the effectiveness of IC in alleviating pain and reducing myofascial tension. However, the literature reports considerable methodological heterogeneity in the application of IC in patients with MTrPs. This heterogeneity is a major obstacle in conducting meta-analyses and systematic reviews [16,17,18], which are essential for developing optimal, evidence-based, and reproducible therapeutic protocols. IC is widely used by therapists due to its simplicity. Nevertheless, there is a lack of high-quality clinical studies that would allow for a comprehensive understanding of the physiological mechanisms underlying IC and, consequently, for the formulation of optimal treatment parameters.

### Aim of the Study

The purpose of the study was to determine the optimal ischemic compression (IC) parameters and to assess the direct and short-term effects of this therapeutic procedure in reference to pain and biomechanical properties in patients with MTrPs in upper trapezius muscles. Hypothesis: IC performed with constant compression force corresponding to the PPT and time indispensable for adaptation of mechanoreceptors effectively elevates the pain sensation threshold of upper trapezius muscle MTrPs.

## 2. Material and Methods

### 2.1. Participants

The study was conducted between February 2024 and April 2024 at the Didactic and Scientific Centre of Warsaw Medical Academy of Applied Sciences in Warsaw, Poland. Its protocol was approved by the Bioethics Committee at the Medical University of Mazovia in Warsaw, Poland (approval reference number: 2022/09/MUM-01) on 30 September 2022. 

A total of 79 of the 81 individuals (56 males and 23 females) qualified for the study. Two participants were excluded from the study due to their voluntary resignation. The inclusion criteria comprised latent MTrPs diagnosed in the course of the qualifying examination, detected in the descending part of the trapezius muscle, with typical symptoms described in [1] and lasting for at least three months; participants’ age range of 20–40 years; and moderate level of physical activity. Exclusion criteria were as follows: age outside the age range constituting inclusion criterion, coexistence of musculoskeletal disorders including systemic ones, previous surgical procedures in the area of the spine and shoulder girdle, intake of analgesics, and recent neck or shoulder injury.

**Figure 1:**
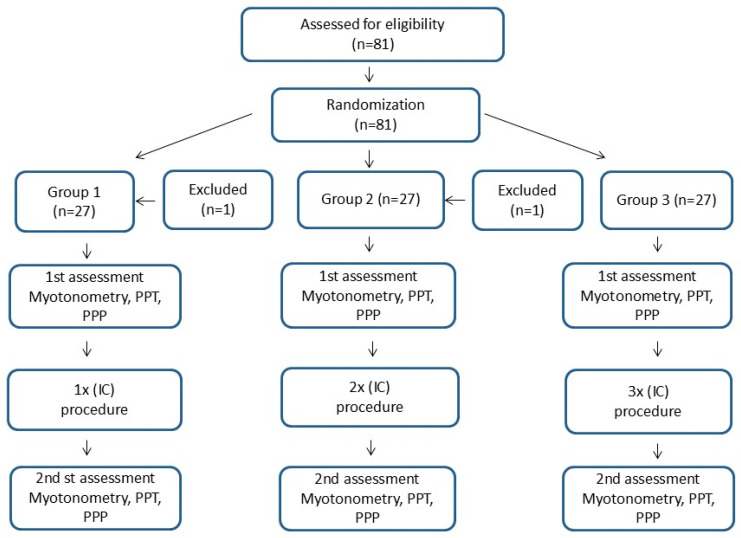
Study design.

After the preliminary qualification, the study participants were randomly assigned to 3 study groups (27 participants in each group), which differed in terms of protocol for performing the IC procedure. One of the authors performed the group assignment using a simple 1:1:1 randomization with a random sequence, using the website randomizer.org. The numbers were given to the participants in sealed envelopes. After randomization, each patient was assigned to specific study group. Each participant gave informed consent in writing to participate in the study. The study design is presented in Figure 1.

### 2.2. Procedures

Assessment of pressure pain threshold (PPT) and pressure pain perception (PPP).

PPT and PPP are commonly used measures within quantitative sensory testing paradigms. PPT and PPP measurements are typically repetitive in nature. During testing for PPT and PPP, the patients were steadily seated, relaxed in a chair with full body support, hands in lap, looking straight ahead. PPT was assessed with the use of a handheld mechanical pressure algometer (Trigger Plus, Palpatronic, Hagen, Germany). The device probe was applied at the angle of 90 degrees, to the surface of descending part of the trapezius muscle, exactly within the projection of MTrP localized earlier by palpation, which was marked on the skin. Pressure was applied at a rate of 10 N/cm^2^/s until the participant first reported a painful sensation. The lowest pressure force at which the patient experienced pain was taken as the value of the measurement (whenever the sensation of pressure was replaced by a sensation of pain). This procedure has shown good intra-rater reliability, with ICC values of 0.64 and 0.96 [19] and from 0.74 to 0.89 [20]. During the PPT measurements, participants were also requested to assess the PPP intensity by means of a visual analogue scale (VAS). This is a scale from 0 to 10, with a 10 cm long graphic section on the other side of the ruler, on which the patient is requested to indicate the intensity of the pain experienced. After inverting the ruler, it was possible to collect the readings, where 0 signifies the absence of pain and 10 represents maximum pain levels. The VAS scale is a reliable and valid measurement tool for assessing pain intensity in the clinical research area [21]. The correlation coefficients of VAS pain scores ranged from 0.64 to 0.84 [22].

#### 2.2.1. Myotonometric Measurements of Muscle Mechanical Properties

Myotonometric measurements were conducted with the use of a MyotonPro device (MyotonPRO, Tallinn, Estonia). Patients were steadily seated in relaxed position on a chair with full body support provided, with hands in lap and looking straight ahead. The device probe was applied exactly within the projection of the MTrP localized earlier, marked on the skin by means of a marker. During automatic calibration, the probe exerted initial pressure on the examined tissue, using a force of 0.18 N. In the subsequent stage, the Myoton PRO device generated a short mechanical impulse 5 times, applying the force of 0.4 N and lasting 15 ms each time, in order to determine the degree of deformation of the examined tissue. The device’s readiness for measurement was signaled by the color of the control diode changing to green. The following parameters of muscular tissue were determined: (a) tone in [Hz]: frequency of oscillations of skeletal muscle, which characterized the muscle tone; (b) dynamic stiffness in [N/m], that is, the muscle’s ability to resist shape changes caused by an external force; (c) logarithmic decrement [log]: attenuation that inversely describes the elasticity of the muscle or the ability to regain its original shape. The results of measurements were interpreted in the following way: the higher the value of tension [Hz] and dynamic stiffness [N/m], the greater the tension and stiffness of the examined tissue; the lower the value of decrement [log], the lower the mechanical energy dissipation during oscillation and the greater the elasticity of the tissue [13,14]. The results of other studies revealed good intra-rater and inter-rater reliability of the MyotonPro device in terms of frequency and stiffness, with intra- and inter-rater ICC values ranging from 0.79 to 0.97. The decrement parameter showed comparatively lower reliability, particularly in inter-rater assessments, with ICC values of 0.65 and 0.93 [23,24].

#### 2.2.2. Ischemic Compression (IC) Procedure

Patients were seated in a steady and relaxed position on a chair, with full body support, hands in lap, and looking straight ahead. The MTrP was located by palpation, performed on both sides. The therapy was applied to the single most painful MTrP. The therapist manually compressed the MTrP, gradually increasing pressure until the patient reported the sensation of pain (up to the level where the sensation of pressure was replaced by a sensation of pain). Compression of the same strength was maintained continuously until the patient reported a significant reduction of pain (conventionally not less than 75%) [25,26,27]. In addition, the time required for the adaption of mechanoreceptors and until the subject reporting that pain was no longer experienced was also measured.

#### 2.2.3. Protocols in Individual Groups

Before starting the study, each participant was informed about the way in which it was to be conducted, as well as about the possibility of experiencing discomfort during its course.

In each of the 3 study groups, before the execution of the therapeutic procedure (IC), the following were conducted, in sequence: myotonometric measurement and PPT together with the assessment of PPP, in order to examine the biomechanical properties of muscle fibers in the MTrP area, pain threshold, and intensification of pain. Subsequently, the IC procedure was performed in respective groups, in accordance with the following protocol:

In group 1—when performing the IC procedure, the compression of MTrP was maintained with constant force for the time required for the pain to subside.

In group 2—when performing the IC procedure, the compression of MTrP was maintained with constant force for the time required for the pain to subside; then, without releasing the compression, its strength was increased until pain was felt again, and once more the compression was maintained with constant force for the time required for the pain to subside.

In group 3—when performing the IC procedure, the compression of MTrP was maintained with constant force for the time required for the pain to subside, which was followed by a progression similar to that applied in group 2. In addition, the compression force was increased yet again, until pain was experienced again, and once more the compression was maintained with constant force for the time required for the pain to subside.

To sum up: in group 1, when performing IC, the adaptation of mechanoreceptors was achieved once, in group 2—twice, and in group 3—thrice. In each of the three study groups, after the completion of the respective IC therapeutic procedure and 5 min of intermission, the following were performed, in sequential order: second myotonometric measurement and (PPT) assessment, together with the assessment of PPP, in order to examine the biomechanical properties of muscle fibers in the MTrP area as well as determine the pain threshold and the intensification of pain.

All the measurements taken by means of the MyotonPRO device as well as the IC, PPT and PPP procedures were performed by one experienced physiotherapist, trained in the field of myotonometry and with knowledge of performing scientific research.

### 2.3. Statistical Analysis

The statistical analysis of the collected data was performed using the Statistica 13 package (Statsoft, Kraków, Poland). The Shapiro–Wilk test was used to test the normality of data. The results are presented as mean values and standard deviation. The Wilcoxon test was used to test the statistical significance of the differences in the examined parameters, before and after the treatment applied (for two matched samples). The statistical significance of differences between the unmatched data (groups) was tested using the Kruskall–Wallis test. The level of statistical significance was set at *p* < 0.05. The effect size (r for Wilcoxon test) was calculated, with r = 0.1 indicating a small effect, r = 0.3 indicating a medium effect, and r = 0.5 indicating a large effect. The effect size (η^2^ for Kruskall–Wallis test) was also calculated, with η^2^ = 0.01 indicating a small effect, η^2^ = 0.06 indicating a medium effect, and η^2^ = 0.14 indicating a large effect. G*power software (version 3.1.9.7; Heinrich—Heine—Universität Düsseldorf, Düsseldorf, Germany; (http://www.gpower.hhu.de, accessed on 25 February 2025) [28] was used to determine the power using 2-sided testing, α = 0.05, with the sample size = 79. The result used for power analysis was tone [Hz]. The effect size was 0.38. The power (1-β err prob) was calculated as 0.91. The test family was “*t*-test”, and the statistical test was the Wilcoxon signed-rank test (marched pairs). The type of power analysis was “Post hoc: Compute achieved power—given α, sample size, and effect size”.

## 3. Results

Individual study groups did not differ with statistical significance as to their size, average age of participants, or average Body Mass Index (BMI) value. In group 1 (n = 26), the average age was 34.57 ± 2.99 years, in group 2 (n = 26) it was 33.61 ± 2.75 years, and in group 3 (n = 27) it amounted to 34.4 ± 3.16 years (*p* = 0.935). The average BMI value in group 1 was: 24.89 ± 2.31 (kg/m^2^), in group 2 it was 25.53 ± 3.29 (kg/m^2^), while in group 3 it was 24.46 ± 3.23 (kg/m^2^); (*p* = 0.906).

The values of the examined parameters for all groups, before and after the ischemic procedure, are presented in Table 1.

**Figure 2:**
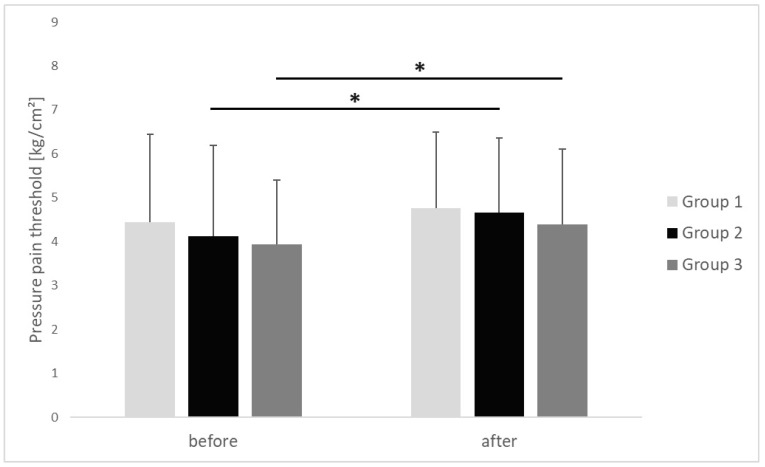
Changes of pressure pain threshold of the upper trapezius trigger points (mean value ± SD) before and after ischemic compression in particular groups. *p* value of <0.05 is represented as *.

The PPT of the upper trapezius MTrP increased in all study groups after performing the IC procedure. In group 1, the average value of PPT of the upper trapezius trigger point MTrP after performing IC increased from 4.44 ± 2.00 kg/cm^2^ before IC started to 4.76 ± 1.72 kg/cm^2^ after the procedure was completed, with the difference close to statistical significance (*p* = 0.053, r = 0.43). In group 2, the average value of PPT of the upper trapezius MTrP increased with statistical significance, from 4.12 ± 2.06 kg/cm^2^ before commencing the IC procedure to 4.66 ± 1.69 kg/cm^2^ after completing the procedure (*p* = 0.009, r = 0.53). In group 3, the average value of PPT of the upper trapezius MTrP also increased with statistical significance, from 3.94 ± 1.45 kg/cm^2^ before the IC started to 4.38 ± 1.72 kg/cm^2^ after completion of the procedure (*p* = 0.009, r = 0.44). We observed a large-size effect in group 2 and medium-size effect in groups 1 and 3. There were no inter-group differences in the PPT of the upper trapezius MTrP before (*p* = 0.703, η^2^ = 0.01) or after the applied procedure (*p* = 0.574, η^2^ = 0.02), and observed effect sizes were small (Figure 2).

In total, in groups 1, 2, and 3, there were 79 procedures of IC performed to the first pain relief reported (first adaptation). The time indispensable for adaptation of mechanoreceptors was measured and averaged 17.0 ± 7.10 s. In groups 2 and 3, a total of 53 IC procedures were performed to the second reported pain subsidence (second adaptation). The time required for adaptation to increased compression, measured from the first subsidence of pain to the second subsidence of pain sensation, amounted to the average of 9.0 ± 3.36 s. The difference observed between the time required for adaptation of mechanoreceptors to the first and second compression force maintained at constant intensity when performing the IC procedure was statistically significant, and observed effect size was large (*p* < 0.001, r = 0.78). In group 3, 27 IC procedures were performed, during which the compression force exerted on upper trapezius MTrP was increased three times.

The time required for adaptation, which was measured from the moment when pain subsided for the second time to the moment it subsided for the third time, amounted to 7.0 ± 2.71 s on average. The difference noted between the time required for the adaptation of mechanoreceptors and the moment when the compression force was applied and maintained at constant intensity for the second and third time during the execution of the IC procedure was not statistically significant, and the observed effect size was small (*p* = 0.227, r = 0.26).

**Figure 3:**
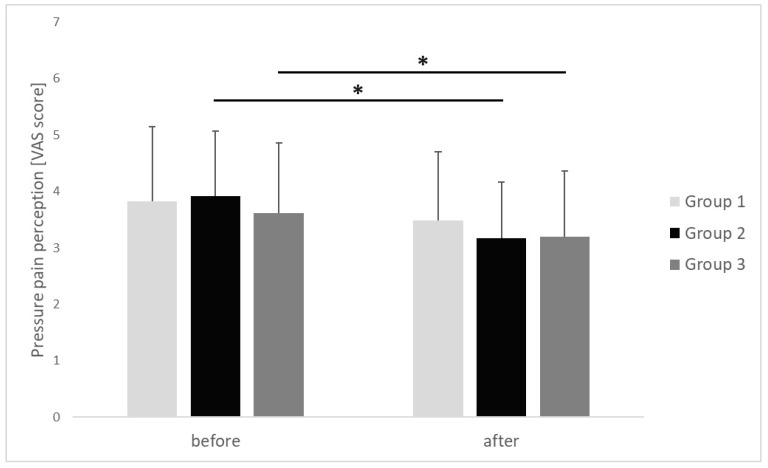
Changes in pressure pain perception of the upper trapezius trigger points before and after ischemic compression in particular groups. *p* value <0.05 is represented as *.

The PPP of the upper trapezius MTrP after performing the IC procedure was reduced in all study groups. In group 1, the average value of PPP of the upper trapezius MTrP after the execution of IC was reduced from 3.82 ± 1.33 [cm] before commencing the IC to 3.49 ± 1.21 [cm] after completion of the procedure, with no statistical significance and a small effect size noted (*p* = 0.623, r = 0.24). On the other hand, in group 2, the average value of PPP at the upper trapezius MTrP decreased statistically significantly, from 3.92 ± 1.15 [cm] before the commencement of the IC procedure to 3.17 ± 1.00 [cm] after procedure completion. Moreover, we observed a large-size effect (*p* = 0.016, r = 0.56). In group 3, the average value of PPP at the upper trapezius MTrP was also statistically significantly reduced, from 3.62 ± 1.24 [cm] before commencing the IC procedure to 3.20 ± 1.16 after performing the procedure (*p* = 0.041, r = 0.48). There were no inter-group differences in the PPP at the upper trapezius MTrP before (*p* = 0.685, η^2^ = 0.02) or after the applied procedure (*p* = 0.761, η^2^ = 0.01) (Figure 3). The observed effect sizes were small.

**Figure 4:**
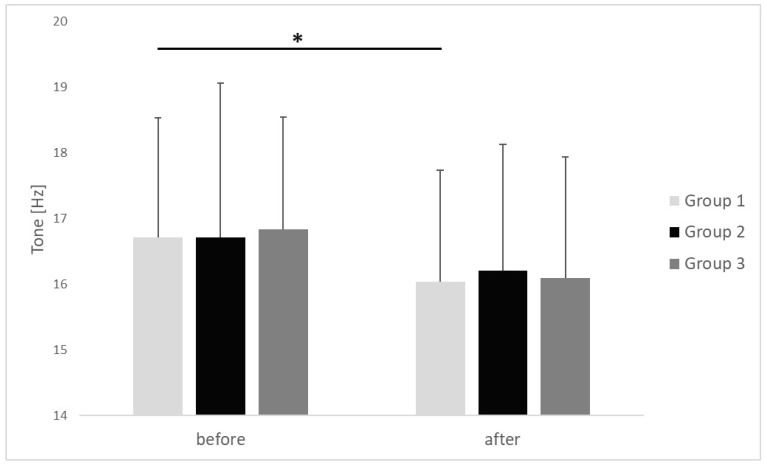
Changes in tone of the upper trapezius trigger points before and after ischemic compression in particular groups. *p* value <0.05 is represented as *.

**Figure 5:**
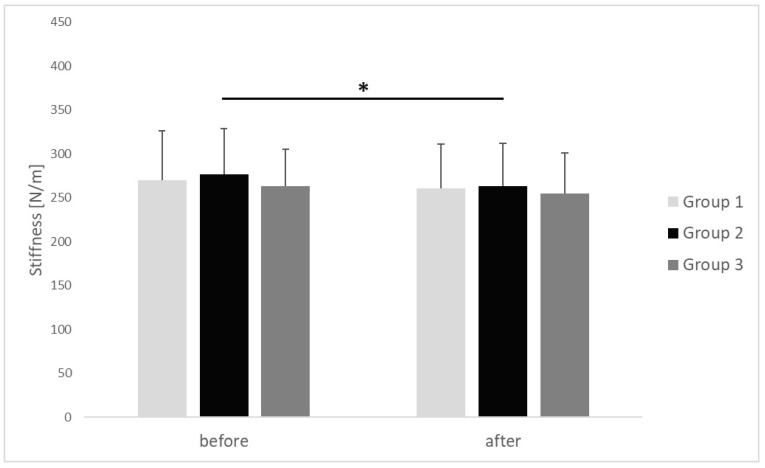
Changes in stiffness of the upper trapezius trigger points before and after ischemic compression in particular groups. *p* value <0.05 is represented as *.

**Figure 6:**
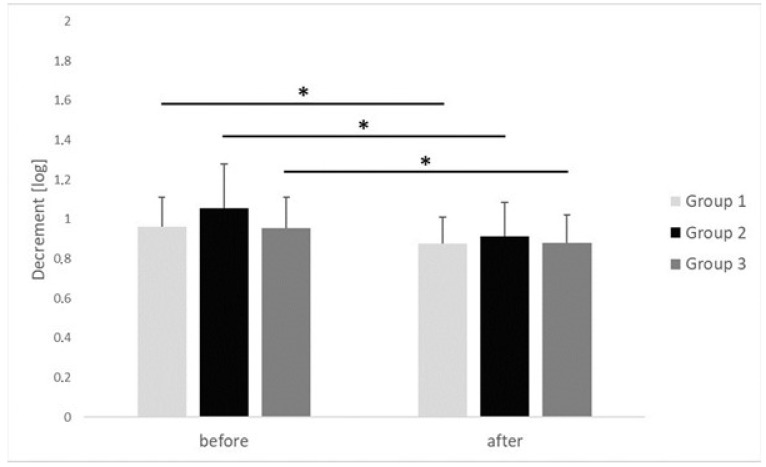
Changes in decrement of the upper trapezius trigger points before and after ischemic compression in particular groups. *p* value <0.05 is represented as *.

The results of myotonometric measurements taken in respective groups before performing the IC procedure and after its completion are presented in Figure 4, Figure 5 and Figure 6.

In group 1, the average value of the tone in the upper trapezius MTrP was diminished, with statistical significance and a large effect size, from 16.71 ± 1.82 Hz before commencing IC to 16.03 ± 1.70 Hz after completion of the procedure (*p* < 0.001, r = 0.66). In groups 2 and 3, the tone at upper trapezius MTrP after completion of the IC procedure demonstrated a decreasing trend, but the differences in relation to the baseline values obtained before the commencing of the procedure revealed no statistical significance (*p* > 0.05). There were no inter-group differences in tone of the upper trapezius MTrP before (*p* = 0.912, η^2^ = 0.02) or after (*p* = 0.856, η^2^ = 0.01) the applied procedure (Figure 4). The observed effect sizes were small.

In group 2, the average value of stiffness in upper trapezius MTrPs decreased with statistical significance and medium effect size, from 276.46 ± 52.43 N/m before the start of IC to 263.15 ± 48.49 N/m after completion of the procedure (*p* = 0.036, r = 0.41). In groups 1 and 3, the stiffness in upper trapezius MTrPs after completion of the IC procedure showed a decreasing trend; however, the differences in comparison with initial values from before the beginning of the procedure turned out to have no statistical significance (*p* > 0.05). There were no inter-group differences in stiffness of the upper trapezius MTrPs before (*p* = 0.924, η^2^ = 0.01) and after (*p* = 0.821, η^2^ = 0.02) the applied procedure (Figure 5). The observed effect sizes were small.

In the case of all groups, the decrement value, which conversely described flexibility, decreased statistically significantly after the IC procedure was performed, in comparison with the initial values measured before the procedure started. In group 1, the average value of decrement after the IC procedure had been performed turned out to decrease, from 0.96 ± 0.15 log to 0.87 ± 0.13 log (*p* = 0.022, r = 0.61). In group 2, the average value of decrement after the IC procedure had been performed decreased from 1.05 ± 0.22 log to 0.91 ± 0.17 log (*p* = 0.001, r = 0.64). We observed large-size effect in group 1 and in group 2. In group 3, in turn, the average value of decrement after the IC procedure had been performed decreased from 0.95 ± 0.15 log to 0.88 ± 0.14 log (*p* = 0.042, r = 0.38). There were no inter-group differences in decrement of the upper trapezius MTrPs before (*p* = 0.882, η^2^ = 0.02) or after (*p* = 0.709, η^2^ = 0.01) the applied procedure (Figure 6). The observed effect sizes were small.

## 4. Discussion

In the study carried out, assessment was made concerning the influence of IC with varying compression levels and procedure duration, depending upon individual pain sensitivity and the adaptation of mechanoreceptors to pain, as well as biomechanical properties of the upper trapezius MTrPs, in immediate evaluation. The most crucial findings were as follows: the PPT of the upper trapezius MTrPs increased significantly after performing the IC procedure in study groups 2 and 3, which was also accompanied by a significant reduction in PPP. PPT increased most significantly in group 3, while PPP was most significantly reduced in study group 2.

On the other hand, no significant differences between groups were observed in the case of PPT and PPP after performing the IC procedures. The indispensable time for the adaptation of mechanoreceptors was significantly shortened already after the first IC procedure. Increasing the compression power during subsequent IC procedures did not result in a significant reduction of the time needed for adapting to the stimulus. Tone and stiffness decreased, while elasticity increased after the IC procedure, in the case of all study groups. Significant reductions were noted in the case of tone in group 1 and stiffness in group 2, while a considerable increase of elasticity was noted in all study groups.

PPT and PPP are useful in conducting a quantitative assessment of MTrP sensitivity and for planning therapies with the application of IC. Manual therapy with the application of IC brings about immediate relief in myofascial pain. The results of the study conducted by Oliviera-Campelo et al. indicate that a single IC procedure performed on upper trapezius MTrP results in increased PPT and reduced PPP, as well as improved range of motion in the cervical spine area. The durability of effects was checked after 24 h and after 1 week. Only the IC procedure, among those performed, namely the Muscle Energy Technique (MET) and Passive Stretching (PS), showed durability of the effect obtained [15]. Those results are consistent with the results reported by Aguilero et al. and by Fernandez-de-las-Penas et al. who, in similar studies, pointed to immediate results concerning the reduction of susceptibility (increase of PPT) in the upper trapezius MTrP after the IC procedure [29,30].

The authors of study [27] assessed the immediate effect of ischemic IC on cervical myofascial pain and trigger-point sensitivity in upper trapezius MTrP. The results of that study indicated that pain threshold values and pain tolerance values were significantly increased after IC procedures had been performed, with force applied until the pain threshold was reached and with a duration of 90 s, and with force applied until the average value of pain threshold was reached and pain tolerance was achieved, in the case of procedure duration times of 30 s, 60 s, and 90 s.

The level of pain intensity assessed using the VAS scale was significantly lower after each IC procedure performed, regardless the force applied and the specific procedure time. IC procedures performed with higher compression force in comparison with a lower pain threshold resulted in markedly higher pain thresholds and pain tolerance values. Additionally, the compression time of 90 s also resulted in significantly better effects in the form of higher pain thresholds and pain tolerance, in comparison with the compression time of 30 s and 60 s. Likewise, a compression time of 90 s resulted in the lowest values of pain intensity assessed using the VAS scale.

As an effective pain alleviation method in the case of pain related to MTrPs in the upper trapezius area, the study authors recommend performing IC procedures either with the pressure applied until the pain threshold is reached, with a long duration of 90 s, or with the pressure applied until the average pain threshold and pain tolerance is reached, with a duration of 30 s [27]. Our results do not fully confirm the above. We demonstrated in the study reported here that after adaptation of mechanoreceptors to the stimulus, further increase of the force applied or extension of the application time does not result in enhancing the analgesic effect.

In our study, the average PPT value for all study subjects was 4.17 ± 1.84 kg/cm^2^ before treatment, and the average PPT value after IC procedures amounted to 4.60 ± 1.68 kg/cm^2^. The obtained values of PPT are higher in comparison with the results obtained by Hou et al., which respectively amounted to: 3.16 ± 0.30 kg/cm^2^ before the IC procedure and 3.76 ± 0.33 kg/cm^2^ after performing the procedure, which might have been expected, as the authors studied people with active upper trapezius MTrPs, and thus the tenderness to palpation on physical examination was higher. The authors also demonstrated a strong correlation of PPT and PPP. The above findings confirmed that these indicators are consistent as regards the assessment of clinical sensitivity of MTrPs and are useful in assessing the therapeutic efficacy of IC in pain relief [27].

Pain relief achieved by IC treatment may result from reactive hyperemia in the MTrP region, counter-irritant effects or a spinal reflex mechanism for the relief of muscle spasm [7,8,10]. The reflex nature of the relaxation of muscle fibers in the MTrP area is also confirmed by the results of the myotonometric measurements reported in our study, which indicate a decrease of tone and stiffness parameters and increased elasticity after the IC procedure.

Janczarzyk et al. demonstrated that a series of five procedures with the use of IC performed until the pain subsided completely and for no longer than 90 s effectively reduces the pain caused by latent MTrP in the upper trapezius area in individuals with UCS. However, on electromyography EMG, they did not observe changes in muscle tone at rest that would exceed physiological norms [31].

Results consistent with our results were reported in the study of Kisilewicz et al., who presented the measurements of muscle stiffness of the trapezius muscle with MTrPs (by means of myotonometry, directly before and after application of single IC). The procedure was performed until a 50% pain subsidence—assessed subjectively—was reported; the length of procedure did not exceed 2 min. A substantial reduction of stiffness in that upper trapezius muscle was noted [13]. In another study, IC therapy comprising seven sessions of therapeutic procedures with a 3-day break, performed in the modality of 5 s of compression and 2–3 s of intermission applied to upper trapezius MTrPs for up to two minutes in a single procedure, caused reduction of tone and stiffness of the upper trapezius muscle, with simultaneous alleviation of pain in muscle, which is similar to what we report in our study [14].

The compression force applied to MTrPs when performing the IC procedure should be within the limit of pain that is tolerated by the patient. Exceeding that limit should be avoided, as this may expose the patient to involuntary autonomic reactions and spinal cord reflexes, resulting in defensive muscle tone. On the other hand, treatment may not be effective if insufficient compression force is used, from the range below PPT. That is why an adequate selection of parameters for an IC procedure is important for securing clinical efficiency. Such a selection is also hindered by inter-subject variability and subjective perception of pain, as well as assessment of pain intensity. This is confirmed by high levels of standard deviation in reference to the average time required for the occurrence of mechanisms of receptor adaptation to the stimuli, as measured in the study reported herein.

According to the study [31], a single IC procedure executed with a constant compression force until the pain subsides completely and for no longer 3 min, applied to latent MTrP in the trapezius muscle, is characterized by greater clinical usefulness in the treatment of pain and increased muscle tone as assessed by (EMG), in comparison with the pulsation mode of performing IC, where the compression force is subject to incessant changes. This finding is in line with the physiological mechanism of the adaptation of receptors to the acting stimuli. The response of receptors to a stimulus of constant intensity decreases with time. In such cases, a significant decrease or receptor potential generation of response may even disappear when a stimulus of constant intensity is applied for a sufficiently long time. As a result, afferent conduction of the nerve impulses responsible for the reduction of pain sensation is weakened [32]. In the IC procedure, a mechanical stimulus is used that affects rapidly adapting (phase-related) mechanoreceptors. That is why the time needed for their adaptation is not long. Thus, after reaching the adaption objective, further prolongation of the IC procedure time no longer results in a significant strengthening of the analgesic effect, which has been fully confirmed by the results of our research.

The results of a meta-analysis of 15 studies with the participation of 725 individuals with pain in cervical muscles indicate IC as the procedure that may be recommended for the immediate and transitory alleviation of pain [33]. Similar conclusions were drawn by the authors of a systematic review [18], who nevertheless advise caution in its application, pointing out the limited quality of certain studies, as well as the absence of the standardization of application parameters of the IC procedure, the existence of which may simplify its applicability [17].

Another meta-analysis [16], which took into account 11 studies involving 427 patients, indicates a substantial increase of PPT after IC procedures in comparison with controls. In the same meta-analysis, comprising seven studies and 251 subjects, no significant difference in comparison with the control group after treatment by means of IC was demonstrated as regards pain intensification measured in VAS analogue scale, concluding that IC merely increases pain tolerance PPT. The authors of the study reported here found neither meta-analyses nor systematic reviews in the literature that assess the results of therapies with the application of IC to muscle MTrPs on the basis of myotonometric measurements.

### Study Limitations and Strengths

The main objective was to examine the influence of procedure parameters (compression force and procedure duration) on the efficacy of therapy. This is the reason why the therapeutic procedure was performed in all subjects, in a manner and under conditions as similar as possible. Further studies are required, as it is not known what number and frequency of ischemic compression procedures is needed to obtain the accumulation of a short-term yet fully repetitive therapeutic effect (based on physiological response). The performance of an analogous study on active trigger points is justified, yet it requires rigorous inclusion criteria concerning the self-assessment of pain. Other study limitations are as follows: the absence of long-term follow-up to assess the persistence of improvements, the absence of a control or sham group, and no blinding of participants or assessor. We recommend conducting a similar study with a larger sample size, which would analyze, in addition to biomechanical parameters, the viscoelastic parameters (stress relaxation time and creep).

The study offers a novel intervention application that is commonly employed in therapeutic environments but has not been studied in a research environment.

## 5. Conclusions

IC performed with constant compression force corresponding to the value of PPT or slightly higher effectively elevates the pain sensation threshold after a single IC procedure, making it possible—in repetitive manner—to achieve the immediate and transient reduction of pain intensity, while the repetition of compression leads to enhancing the therapeutic effect. The manner of performing the therapeutic procedure that has been presented also leads to the improvement of biomechanical parameters of muscle fibers in the trigger point area, without causing significant discomfort to the patient.

## Figures and Tables

**Table 1:** Summary of the examined parameters before and after the ischemic compression procedure, with corresponding *p*-values and effect sizes for all groups.

		Group 1	Group 2	Group 3		
		Mean ± SD	Mean ± SD	Mean ± SD	*p* **	η^2^
Pressure pain threshold [kg/cm^2^]	before	4.44 ± 2	4.12 ± 2.06	3.94 ± 1.45	0.703	0.01
	after	4.76 ± 1.72	4.66 ± 1.69	4.38 ± 1.72	0.574	0.02
	*p* *	0.053	0.009	0.009		
	r	0.43	0.53	0.44		
Pressure pain perception [VAS score]	before	3.82 ± 1.33	3.92 ± 1.15	3.62 ± 1.24	0.685	0.02
	after	3.49 ± 1.21	3.17 ± 1	3.2 ± 1.16	0.761	0.01
	*p*	0.623	0.016	0.041		
	r	0.24	0.56	0.48		
Tone [Hz]	before	16.71 ± 1.82	16.72 ± 2.34	16.84 ± 1.71	0.912	0.02
	after	16.03 ± 1.7	16.21 ± 1.93	16.10 ± 1.84	0.856	0.01
	*p*	<0.001	0.082	0.068		
	r	0.66	0.51	0.69		
Stiffness [N/m]	before	270.31 ± 56.29	276.46 ± 52.43	263.33 ± 42.29	0.924	0.01
	after	260.92 ± 50.36	263.15 ± 48.49	254.67 ± 46.45	0.821	0.02
	*p*	0.059	0.036	0.071		
	r	0.34	0.41	0.29		
Decrement [log]	before	0.96 ± 0.15	1.05 ± 0.22	0.95 ± 0.15	0.882	0.02
	after	0.87 ± 0.13	0.91 ± 0.17	0.88 ± 0.014	0.709	0.01
	*p*	0.022	0.001	0.042		
	r	0.61	0.64	0.38		

*p* *—Wilcoxon test; *p* **—Kruskall–Wallis test; η^2^—effect size.

## Data Availability

Data are available upon reasonable request as addressed to the corresponding author.

## Abbreviations

MTrPs	Myofacial Trigger Points
IC	Ischemic compression
PPT	Pressure Pain Threshold
PPP	Pressure Pain Perception
ATP	Adenosine Triphosphate
CGRP	Calcitonin Gene-Related Peptide
UCS	Upper Cervical Syndrome
VAS	Visual Analogue Scale
BMI	Body Mass Index
MET	Muscle Energy Technique
PS	Passive Stretching
EMG	Electromyography
